# Greener and Whiter Analytical Procedure for Theobromine and Caffeine Determination in Tea Using Dimethyl Carbonate as an Extraction Solvent and Mobile Phase Constituent in Reversed-Phase Liquid Chromatography

**DOI:** 10.1021/acsomega.4c11625

**Published:** 2025-03-24

**Authors:** Oktawia Kalisz, Martina Catani, Szymon Bocian

**Affiliations:** † Department of Environmental Chemistry and Bioanalytics, Faculty of Chemistry, Nicolaus Copernicus University, 7 Gaga-rin St., Toruń 87-100, Poland; ‡ Department of Chemical, Pharmaceutical and Agricultural Sciences, University of Ferrara, via L. Borsari 46, Ferrara 44121, Italy

## Abstract

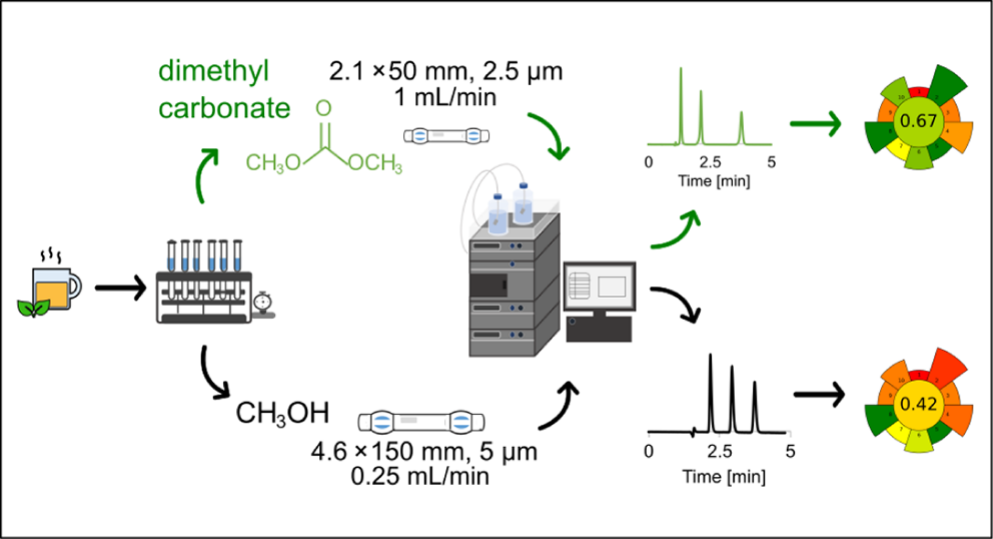

For the first time, dimethyl carbonate was applied as the sole organic solvent, both as an eluent in solid-phase extraction (SPE) and for the simultaneous determination of theobromine and caffeine in tea extracts using reversed-phase ultrahigh-performance liquid chromatography (RP-UHPLC). The proposed procedure has been validated and complies with the requirements of green analytical chemistry (GAC). Solid-phase extraction was used to purify the samples, achieving high recovery rates (97–101%) and precision (RSD < 2%). The method showed satisfactory sensitivity (achieving limit of quantitation values of 0.2 ng for theobromine and 0.35 ng for caffeine), although it was poorer compared to the developed conventional HPLC method using methanol, where the values reached 0.045 and 0.05 ng, respectively. The low toxicity and minimal solvent consumption of DMC contributed to a much more beneficial environmental profile of the developed method, as confirmed by AGREEprep, AGREE, and ChlorTox evaluations. Moreover, by switching from conventional HPLC to UHPLC, the remaining principles of GAC were further incorporated by reducing solvent consumption, analysis time, and energy requirements. In addition, the RGB tool highlighted the higher environmental performance of the method using DMC while not compromising analytical quality or economic practicality. The robustness and environmental benefits make this method a viable alternative for the routine analysis of tea ingredients, supporting sustainable analytical practices.

## Introduction

Demand for sustainable practices in chemical analysis, driven by environmental concerns, has increased significantly in recent decades. This shift is further motivated by growing public pressure, government regulation and global initiatives to combat the alarming consequences of climate change, loss of biodiversity and rising incidence of pollution-related diseases. These interrelated challenges not only threaten ecosystems, but also pose serious risks to public health, underscoring the critical need for more environmentally friendly solutions in all fields of science and industry.^1^

Green chemistry favors the design of chemical products and processes that reduce or eliminate the use and generation of hazardous substances. Its principles, such as waste prevention, use of safer solvents and energy efficiency, provide a framework for sustainable practices in various fields. Green analytical chemistry (GAC) applies these principles to analytical procedures, aiming to minimize the environmental footprint of sample preparation, analysis and disposal.^2−4^ However, GAC focuses on the environmental aspect, overlooking the financial impact or efficiency, which is contrary to the fundamentals of sustainable chemistry. To overcome these disadvantages in 2021, Nowak et al.^5^ proposed the term White analytical chemistry (WAC), which, in addition to the principles of GAC, emphasizes the holistic adjustment of analytical procedures, also taking into account performance reliability and economic aspects.^6^

High-performance liquid chromatography (HPLC) has several significant disadvantages from the point of view of GAC, as it consumes large amounts of energy, involves the use of hazardous organic solvents, generates a large amounts of waste, and is time-consuming which is a significant environmental concern. Some mitigation of these disadvantages is provided by the application of the UHPLC technique, since, in combination with the use of columns with reduced dimensions, it allows for a significant decrease in waste generation and shorter analysis times.^7,8^

Reversed-phase liquid chromatography (RP LC) is a fundamental technique in analytical chemistry, that relies on methanol (MeOH) and acetonitrile (ACN). They owe their widespread use to their low viscosity, high elution strength, UV-transparency, and unlimited miscibility with water.^9,10^ However, prolonged exposure to ACN and MeOH poses significant health risks, including the formation of toxic metabolites like hydrogen cyanide and formaldehyde, as well as damage to the nervous system, liver, and kidneys, raising concerns about their extensive use in LC applications.^11^ To address these challenges, scientists have been exploring alternative solvents among which dimethyl carbonate (DMC) is gaining popularity due to its nontoxicity and biodegradability.^12−14^ DMC in RP LC was first used in the work of Lajin et al.,^15^ where it was used as an organic modifier to separate and detect 11 pharmaceutical molecules, with inductively coupled plasma mass spectrometry (ICP-MS). Due to its higher nonpolarity, and thus elution strength, a lower percentage of DMC was needed to achieve elution of analytes compared to MeOH or ACN. Although DMC shows potential, its application in RP LC remains limited due to its poor miscibility with water (∼10% solution).^9^ This limitation makes it unsuitable for the elution of highly hydrophobic compounds such as polycyclic aromatic hydrocarbons.

Quantification of the sustainability of analytical procedures requires the use of objective metrics. In recent years several new tools have been developed to evaluate and compare the environmental performance of methodologies.^16,17^ Among these, AGREE (Analytical GREEnness Calculator) and AGREEprep are noteworthy; AGREE assess the compliance of analytical methods with the GAC principles and generates a comprehensive greenness score, taking into account factors such as waste, energy consumption, and the use of hazardous substances while AGREEprep specifically evaluates sample preparation methods since it is often the most resource-intensive part of the analytical process.^18,19^ ChlorTox is another metrics that focuses on the toxicity and harmfulness of solvents used in analytical processes, promoting their substitution and minimization.^20^ Complementing these tools is the RGB tool, which integrates environmental, economic, and performance aspects of analytical methods. By visually presenting the balance between these dimensions, the RGB tool guides the method development toward the principles of WAC.^21^ Together, these tools enable researchers to comprehensively evaluate analytical methods for their greenness and efficiency.

Caffeine and theobromine are methylxanthines commonly found in teas, cocoa beans, kola nuts and guarana. While caffeine is widely recognized for its stimulating effects, theobromine contributes to milder physiological effects. The content of these compounds in teas varies depending on tea type, processing, and preparation, with green and black teas typically containing higher levels of caffeine, whereas theobromine content is more variable.^22,23^ Quantitative determination of these compounds is essential for quality control, consumer information, and understanding health impacts.

Traditional methods for analyzing caffeine and theobromine in teas include sample preparation with hot water extraction in volumes typically between 50 – 250 mL followed by filtration.^24,25^ Usually, the next step is solid-phase extraction for purification with organic solvents such as chloroform^26,27^ or acidified methanol,^28,29^ followed by chromatographic separation and identification by HPLC-UV technique. Standard mobile phase constituents are water (sometimes acidified) with methanol,^29,30^ acetonitrile,^25^ or occasionally ethanol.^31^ While effective, these procedures raise environmental and safety concerns due to the use of large solvent volumes and hazardous chemicals. Some example procedures for the determination of these compounds are summarized in Table 1.

**Table 1:** Summary of Example Methods Used for the Determination of Caffeine and Theobromine in Tea (TB – Theobromine, TF – Theophylline, CF – Caffeine

Analytes	Matrix	Sample preparation	Analysis conditions	ref.
TB, TF, CF	Tea and sport drinks, chocolate milk, soft and energy drinks, coffee powder	For tea samples: filtration, pH adjustment to pH 8; cleanup with LC-18 SPE cartridges (conditioning with 12 mL of MeOH and 12 mL of H_2_O, washing with 6 mL of H_2_O, elution with 10 mL of chloroform)	HPLC-DAD; RP-8 column (5 μm, 4.6 × 150 mm) was used with the mobile phase 0.1% THF in H_2_O, pH 8)/ACN (90:10, v/v); 0.8 mL/min; 25 °C, 273 nm; analysis time: 11 min	(26)
TB, TF, CF	Chocolate, coffee, tea, coconut water, human urine	For tea samples: extraction with hot water (2 g in 150 mL); double filtration	HPLC-UV; C18 column (5 μm, 4.6 × 150 mm) was used with the mobile phase (A) MeOH/H_2_O/acetic acid or (B) EtOH/H_2_O/acetic acid (20:75:5, v/v/v); 0.7 mL/min; 273 nm; analysis time: (A) 12 min, (B) 6 min	(31)
TF, CF	Tea samples	Extraction with water at 50 °C (5 g in 150 mL) for 4 h; filtration; cleanup with M-SPE (conditioning with 9 mL of MeOH/acetic acid (90:10, v/v) and 9 mL of MeOH, washing with 1 mL of MeOH, elution with 1 mL of MeOH/acetic acid (90:10, v/v)	HPLC-UV; C18 column (5 μm, 4.6 × 250 mm) was used with the mobile phase MeOH:H_2_O (60:40, v/v), 0.6 mL/min; 270 nm	(28)
TB, TF, CF	Tea samples	Extraction with hot water (2 g in 250 mL or 5 g in 200 mL), centrifugation and dilution of the supernatant	HPLC-PDA; C18 column (3 μm, 4.6 × 100 mm) was used with the mobile phase ACN/H_2_O (90:10, v/v); 1 mL/min; 35 °C, 273 nm; analysis time: 8 min	(25)
TF, TB	Tea samples	UAE with EtOH (ratio of homogenized sample to EtOH 1:20 g/mL) for 1 h, filtration; cleanup with M-SPE cartridges (conditioning with 1.5 mL of MeOH and 1.5 mL of H_2_O, washing with 1.5 mL of MeOH/H_2_O (80:20, v/v), elution with 4 mL of MeOH/acetic acid (80:20, v/v) pH 3	HPLC-UV; C18 column (5 μm, 4.6 × 250 mm) was used with mobile phase MeOH/H_2_O)/acetic acid (20:80:2, v/v/v); 0.8 mL/min; 280 nm; analysis time: 10 min	(29)
TB, TF, CF	Tea samples	Extraction with hot water (100 mg of homogenized sample in 50 mL), centrifugation, addition of supernatant to 15 mg of graphene oxide (GO) sorbent (conditioned with 1 mL of MeOH and 2 mL of H_2_O), sonification, shaking, addition of NaCl, desorption with 100 μL of alkaline MeOH	HPLC-UV; C18 column (5 μm, 4.6 × 250 mm) fitted with C18 guard column was used with the mobile phase MeOH/H_2_O/formic acid (18.7:81:0.3, v/v/v); 1 mL/min; 280 nm; analysis time: 25 min	(24)

This study introduces a novel analytical procedure for the determination of theobromine and caffeine in teas, utilizing dimethyl carbonate as both an eluent in SPE and a mobile phase constituent in reversed-phase UHPLC. This approach is compared to a reference method employing methanol in both extraction and HPLC analysis. The methodologies are evaluated using tools such as AGREE, AGREEprep, ChlorTox, and the RGB model to evaluate their greenness and analytical performance. This study is the first to utilize dimethyl carbonate as the sole organic solvent throughout the entire analytical process. By moving from HPLC to UHPLC, this method also achieves significant reductions in energy consumption, solvent usage, and analysis time, further enhancing its environmental sustainability and efficiency and setting a benchmark for more environmentally friendly practices in liquid chromatography.

## Experimental Section

### Materials and Reagents

Kromasil Eternity C18 (2.1 × 50 mm, 2.5 μm) and Kinetex C18 columns (4.6 × 150 mm, 5 μm) (Phenomenex, Torrance, CA, USA) were applied. HPLC grade methanol was purchased from J.T. Baker (Avantor, Radnor, PA, USA). Dimethyl carbonate was obtained from Sigma-Aldrich (Saint Luis, MO, USA). Water was prepared with a Milli-Q Water Purification System (Millipore Corporation, Bedford, MA, USA). The theobromine, theophylline and caffeine standards were purchased from Sigma-Aldrich (Saint Luis, MO, USA). Polimeric SPE cartridges Strata-X 33 μm (100 mg/3 mL) were purchased from Phenomenex (Torrance, CA, US).

### Apparatus

The chromatographic analyses were conducted using two liquid chromatographs. The first one, Shimadzu Nexera UHPLC system (Tokyo, Japan) is equipped with a binary solvent delivery pump (LC-30AD), an autosampler with a 20 μL volume loop (SIL-20AC), a column temperature controller (CTO20AC), and a diode-array UV–vis detector (SPD-M20A). The second, Shimadzu Prominence HPLC system (Kioto, Japan) includes a quaternary solvent delivery pump (LC-20AD), an autosampler (SIL-20A), a column thermostat (CTO-10 AS VP), and a diode-array UV–vis detector (SPD-M20A). Data acquisition and instrument control were managed using LabSolutions LC/GC 5.65 software developed by Shimadzu (Kioto, Japan).

### Samples

A total of 10 teas with different levels of processing (teas with fully ground leaves and only partially ground leaves) were selected for the study. Among the selected teas, 5 were black and 5 green. All tea samples were purchased from a local supermarket in Toruń, Poland.

### Methods

Both of the proposed methods consist of hot water extraction of tea material and solid phase extraction for sample purification. Further obtained extracts are analyzed by HPLC and UHPLC techniques. Quantitative analysis was performed using the standard addition method by enriching purified tea extracts. In the greener method with DMC, 0, 50, 100 and 150% of the analyte concentration was added by diluting the standard mixture containing 7.5 μg/mL theobromine and 75 μg/mL caffeine. The concentrations of added analytes in the samples were: 0.5; 1.0; 1.5 μg/mL theobromine and 5; 10; 15 μg/mL caffeine. For the reference method, the standard mixture contained 5 μg/mL theobromine and 50 μg/mL caffeine, while the concentrations of added analytes in the samples were: 0.25; 0.50; 0.75 μg/mL theobromine and 2.5; 5.0; 7.5 μg/mL caffeine.

#### Sample Preparation

**Figure 1:**
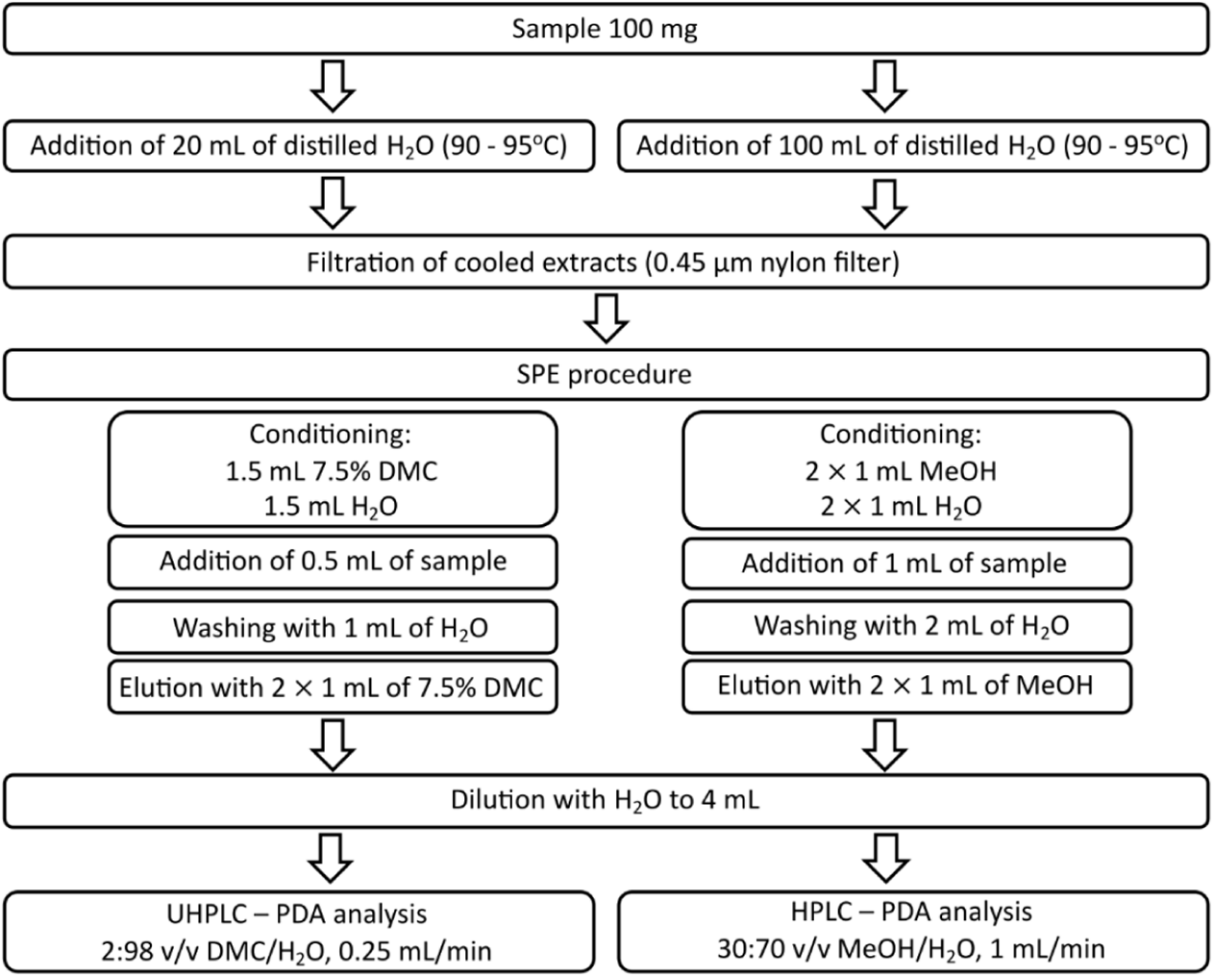
A diagram of both sample preparation procedures; the green one using dimethyl carbonate and the reference one using methanol.

In the case of greener procedure a sample of 100 mg of homogenized tea leaves was taken into a beaker and 20 mL of distilled water was subsequently added. The water temperature was in the range 90–95 °C. After cooling down, the obtained extract was filtered by a nylon filter of 0.45 μm. Commercial tea consists of many components that cause chromatographic interferences with theobromine and caffeine. For this reason, the sample purification consists of SPE with Strata-X cartridges. It enables the separation of both alkaloids and removes most of the interfering components. In a reference procedure, the same sample of 100 mg of homogenized tea leaves was taken into a beaker and 100 mL of hot distilled water was added. After cooling, the resulting extract was also filtered and subjected to the SPE procedure according to the instructions obtained from the SPE cartridges manufacturer.^32^ Detailed procedures are presented in Figure 1.

#### Chromatographic Methods

To demonstrate how analytical procedures can be comprehensively modified to reduce the environmental footprint, in addition to the introduction of DMC as a green solvent, additional GAC principles were applied, including miniaturization, energy efficiency, and solvent savings by transitioning from HPLC to UHPLC. This allowed the application of a column with reduced chromatographic dimensions (2.1 × 50 mm) which decreased the amount of mobile phase consumption compared to conventionally used 4.6 × 150 mm columns. Separation was performed using a binary mobile phase of dimethyl carbonate/water (2/98 v/v) with a flow rate of 0.25 mL/min. The column thermostat was set at 30 °C and the autosampler temperature was set at 5 °C. The injection volume was 1 μL, while detection was performed at 273 nm. The reference method was based on standard HPLC equipment, and separation was performed using a conventional methanol/water mobile phase (30/70 v/v) at a flow rate of 1 mL/min. The column thermostat was set at 30 °C, while the autosampler temperature was set at 5 °C. The injection volume was 10 μL. Detection was performed at 273 nm. Unless otherwise specified, all measurements were performed in triplicate.

#### Methods Validation

Different validation characteristics, including accuracy, precision, linearity, limit of detection (LOD), and limit of quantification (LOQ), were tested to demonstrate the suitability of both green and reference procedures. The limit of detection and quantification were determined experimentally based on the signal-to-noise ratio (LOD = 3 × S/N and LOQ = 10 × S/N). The precision of the analytical procedure was considered at two levels: intraday and interday. For intraday precision, 3 levels of theobromine and caffeine concentrations (low, medium, and high) prepared in tea extracts were injected in triplicate at 3 different times on the same day. For interday precision, samples were injected on 3 different days (1st, third, and seventh) in five replicates. The suitability of the results was determined by calculating the relative standard deviation (RSD).

To determine the recovery for the green procedure, an aqueous solution of theobromine and caffeine at concentrations of 0.1 and 0.5 mg/mL, respectively (and 1 μg/mL and 10 μg/mL, respectively, for the reference procedure), was prepared and the tea extracts were enriched by adding 50, 100 and 150% of the analytes (relative to the unenriched sample). Samples were extracted in 3 replicates at each concentration level.

After determining the analyte content, the recovery was calculated based on the eq 1.
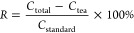
1where R is the recovery rate, *C*_total_ is the total amount of the determined analyte in sample, C_tea_ is the amount of the analyte found in not spiked tea sample and C_standard_ is the amount of added standard solution of analytes.

### Evaluation of Methods’ Greenness and Whiteness

To compare the greenness of the methods presented, tools were chosen that take into account different aspects in order to comprehensively examine them. Therefore, the AGREEprep tool was selected to evaluate the greenness of sample preparation stage. To compare holistically the compliance of the two procedures with GAC principles, the AGREE tool was used. To get the result from the AGREE and AGREEprep calculators, it was necessary to download the free software available at^33^ and enter all the required data.

Since the most significant variable in the proposed greener method is the application of an alternative organic solvent, dimethyl carbonate, the next tool chosen to evaluate the methods was the ChlorTox Scale. This tool allows measuring the harmfulness, toxicity and quantity of reagents used in an analytical method by comparing the substance in question with chloroform as a reference substance.

To compare the whiteness of the methods presented RGB tool was used. The RGB tool provides a systematic approach to assess three key parameters: Red (R) for analytical performance, Green (G) for greenness, and Blue (B) for practicality. It is a highly flexible tool, so it can be tailored to the needs of the user’s laboratory and the specifics of the procedure being evaluated. Four parameters were chosen to evaluate analytical performance: precision, accuracy, linearity range and sensitivity. For ecological evaluation, the focus was on comparing the amount of organic solvents required, their harmfulness, the amount of waste generated and energy consumption. The economic aspect, on the other hand, included a comparison of cost-effectiveness, sample throughput and sample consumption. Detailed values assigned to each parameter are presented in the Supporting Information.

## Results and Discussion

### Method Development and Validation

**Figure 2:**
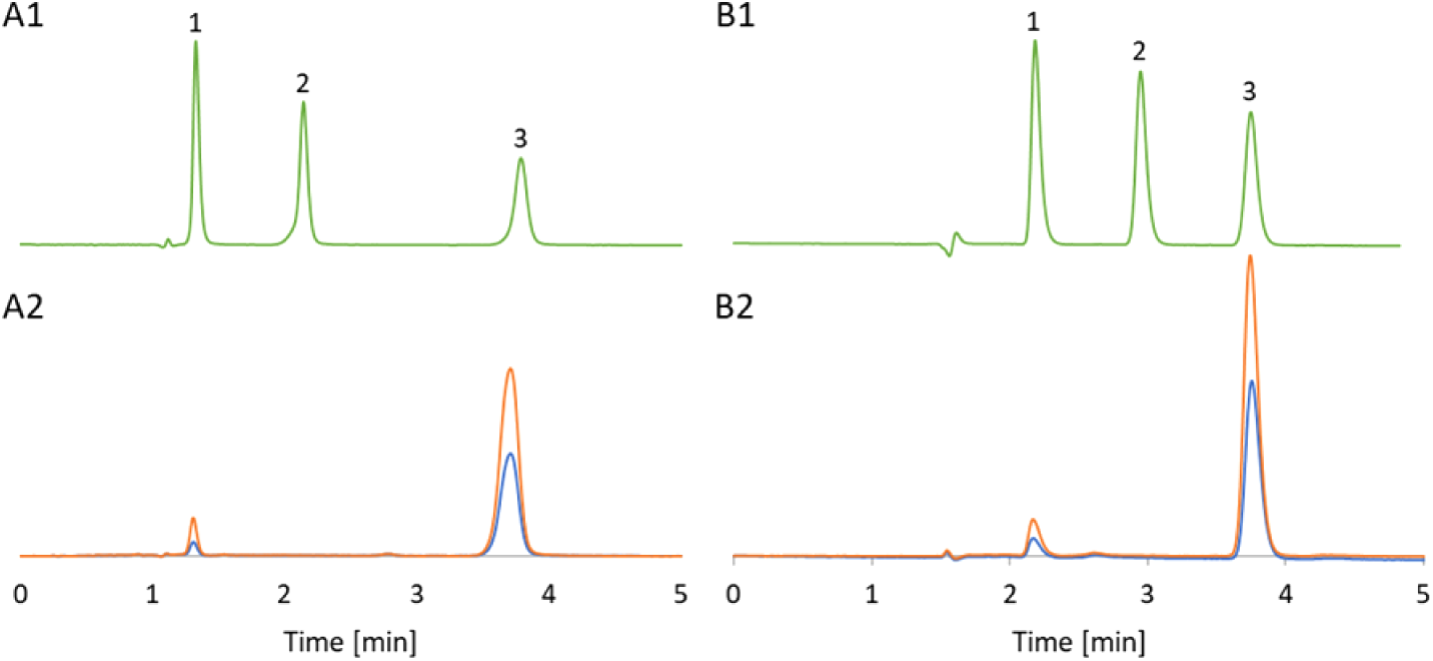
Chromatograms obtained with (A) DMC method and (B) MeOH method of (A1) and (B1) a standard solution of (1) theobromine, (2) theophylline and (3) caffeine; (A2) and (B2) black tea extract without spiking and spiked with standard solution of the analytes (theobromine and caffeine).

A more eco-friendly method for purifying tea extracts by SPE technique using only dimethyl carbonate as the organic solvent and simultaneous determination of theobromine and caffeine using DMC as the mobile phase component was developed. New procedure employing dimethyl carbonate was compared with developed standard method employing methanol. Comparative chromatograms are shown in Figure 2. As can be seen in the chromatograms analysis methods developed also allow the identification of theophylline, but since we could not detect it in the tea samples tested (as in the articles,^25,27^ it is not the subject of the study.

The basic validation parameters were determined for the developed methods: linearity, LOD, LOQ, inter- and intraday precision. Methods are linear over a wide range of analytes concentrations in both methods. Using the regression analysis, the determination coefficients were determined to be at least 0.999 for both methods and analytes. Detailed data are listed in Table 2. The greener method with dimethyl carbonate relies on the UHPLC technique, which generally offers higher sensitivity than HPLC due to sharper peaks and improved resolution. In this study, the most typical injection volumes were applied – 10 μL for HPLC and 1 μL for UHPLC. As a result, the LOD and LOQ demonstrated greater sensitivity for the HPLC method using methanol. Nevertheless, increasing the UHPLC injection volume can easily improve the sensitivity. Comparing the sensitivity of the two methods with the standard method for the determination of these analytes available in the literature,^27^ both of them have sensitivities up to 1 order of magnitude higher. A method with higher sensitivity is also available.^24^ Recoveries of 97–101% obtained for both procedures presented demonstrate the satisfactory efficiency and accuracy of the SPE purification methods. This range indicates minimal analyte loss, ensuring high reproducibility and reliability of the extraction process. This level of recovery confirms that the methods are well suited for quantitative analysis.

**Table 2:** Validation Parameters for Both Developed Methods

	Method with DMC		Method with MeOH	
Parameter	TB	CF	TB	CF
Slope	7.03	6.00	28.43	25.12
Intercept	– 0.35	– 0.58	2.92	2.87
Linearity range [μg/mL]	0.2 – 25	0.35 – 25	0.045 – 25	0.05 – 25
Correlation coefficients (R^2^)	0.9999	0.9999	0.9997	0.9996
LOD [μg/mL]	0.08	0.1	0.02	0.02
LOQ [μg/mL]	0.2	0.35	0.045	0.05

The intraday and interday precision results, with relative standard deviation (RSD) values below 2% also obtained for both methods, show satisfactory repeatability and robustness of the method, confirming its reliability in routine analytical use. Detailed data are presented in Tables 3 and 4.

**Table 3:** Precision and Accuracy Results for Greener Method Employing DMC

	RSD [%]		Recovery		
Compound	Intraday	Interday	Amount added [μg/mL]	Recovery [%]	RSD [%]
Theobromine	0.43	1.80	0.5	98.83	2.38
	0.83	0.89	1	98.96	2.54
	0.61	0.75	1.5	99.34	1.15
Caffeine	0.29	0.37	5	98.40	1.75
	0.44	0.42	10	99.19	2.84
	0.19	0.19	15	98.85	0.19

**Table 4:** Precision and Accuracy Results for Reference Method Employing Methanol

	RSD [%]		Recovery		
Compound	Intraday	Interday	Amount added [μg/mL]	Recovery [%]	RSD [%]
Theobromine	0.51	0.87	0.25	101.37	0.56
	1.66	1.37	0.5	97.29	1.03
	1.71	1.52	0.75	100.49	0.15
Caffeine	0.38	0.43	2.5	100.70	3.42
	0.79	0.75	5	96.61	1.22
	1.20	1.21	7.5	100.56	0.62

### Sample Analysis

Based on satisfactory validation parameters, the new green method was applied to the extraction and quantitative analysis of theobromine and caffeine from two types of tea beverages (black and green teas) to evaluate the applicability of the developed greener method. The results are summarized in Table 5. The obtained content values for caffeine are generally within the typical range for teas, 2 – 5%, while for theobromine they are in most cases slightly below the expected range of 0.15 – 0.20%.^34,35^ Higher contents of both analytes were observed for black teas. In general, studies show that theobromine and caffeine content in teas can vary widely and is usually higher in teas which leaves are in less processed forms.^36^ The results also confirm that the developed method applying the more environmentally friendly solvent, dimethyl carbonate, provides reliable results.

**Table 5:** Theobromine and Caffeine Contents in Analyzed Tea Samples

			Analyte concentration			
			Theobromine		Caffeine	
Tea number	Type of tea	Degree of leaf fragmentation	mg/g	%	mg/g	%
B1	Black tea	fragmented leaves	2.60	0.26	22.89	2.29
B2		fragmented leaves	0.90	0.09	30.73	3.07
B3		granulate	1.28	0.13	18.37	1.84
B4		granulate	1.15	0.12	21.45	2.15
B5		granulate	1.66	0.17	20.98	2.10
G1	Green tea	granulate	1.39	0.14	20.50	2.05
G2		granulate	0.37	0.04	18.36	1.84
G3		fragmented leaves	0.20	0.02	14.23	1.42
G4		fragmented leaves	0.15	0.02	16.13	1.61
G5		granulate	0.99	0.10	21.60	2.16

### Greenness and Whiteness Evaluation of the Developed Methods

**Figure 3:**
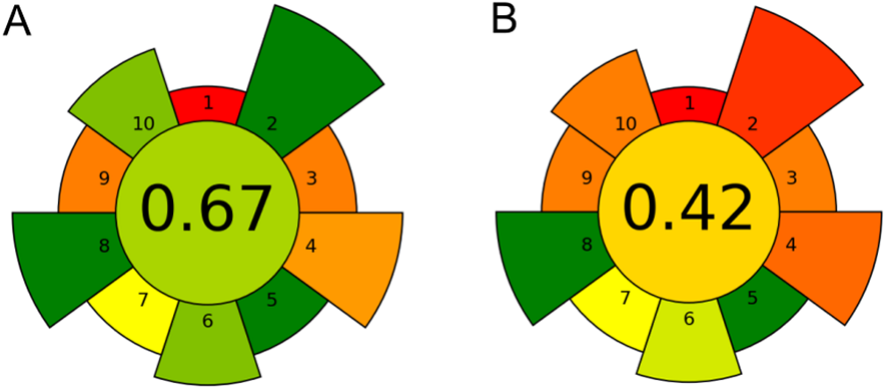
Results of greenness evaluation of the sample preparation stage of the developed methods using (A) DMC; (B) MeOH obtained with the AGREEprep tool.

Based on the results obtained from the AGREEprep software, it can be concluded that the overall greenness assessment of the sample preparation step, taking into account both sample preparation by hot water extraction and purification by SPE technique, performed more favorably for the method using dimethyl carbonate as can be concluded from pictograms shown in Figure 3. Since the compared procedures have several elements in common in the overall sample preparation scheme as well as the subsequent chromatographic analysis, some of the individual criteria evaluated by AGREEprep have the same subrating, such as criterion 1, 3, 5 or 9, which reduces the disproportion between final ratings. However, the simple replacement of methanol with dimethyl carbonate results in a significant improvement in the evaluation of criteria primarily 2 and 10, and the change in apparatus mainly improves the evaluation of criterion 4, classifying this method as green compared to the reference method marked in orange.

**Figure 4:**
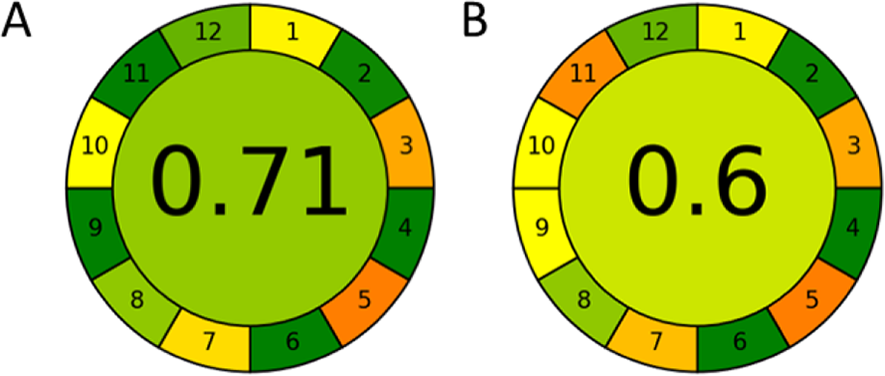
Results of greenness evaluation of the developed methods using (A) DMC; (B) MeOH obtained with the AGREE tool.

The next greenness assessment conducted with the AGREE tool concerns all aspects related to the analytical procedures. As with AGREEprep, some of the subcriteria received identical scores due to the similarity of the methods evaluated consisting of the same sample preparation procedure (criteria 1 and 4) and the use of liquid chromatography in the identification and

determination of the same analytes (criteria 3 and 8). However, this tool positively distinguished the advantages of the UHPLC technique over HPLC (criterion 9). While the replacement of methanol with dimethyl carbonate was positively evaluated in criterion 11. Based on the results obtained from the AGREE tool presented in Figure 4, it can be concluded that the procedure using DMC is greener than the one with MeOH. However, it should be noted that the reference method is greenish. This is due to the fact that the chromatographic analysis is fast (takes 5 min, the same as the method using DMC), and therefore allows the consumption of a relatively small volume of solvents. In the methods available in the literature,^24,26^ the analysis is usually 2 or even 4 times longer which generates correspondingly larger amounts of waste. In addition, only methanol was applied as an eluent to purify the extracts, which is already a greener alternative, since equally common eluents for these analytes are chloroform and dichloromethane which are carcinogenic compounds.^24,27,37^

Since the main variable in the compared methods was the application of different organic solvents, the ChlorTox tool was used to assess the environmental performance. The tool took into account the categories of harmfulness of these solvents assigned to them in their safety data sheets, the mass of solvents used required to prepare and analyze one sample, and compared the resulting values to chloroform as a reference. The only harmfulness of dimethyl carbonate is its flammability, in addition, due to its higher nonpolarity, it has a higher elution strength in a reversed-phase system, so its consumption is low. Unlike methanol, which has been assigned 5 different categories of harmfulness related to flammability and toxicity. In addition, methanol has a relatively low elution strength, so both at the solid phase extraction step and during the analysis, it was necessary to use it in larger amounts. Summarizing these factors, the ChlorTox value for method employing DMC was 0.4 and for method using MeOH was 2.46, indicating that DMC allows achieving a greener procedure.

**Figure 5:**
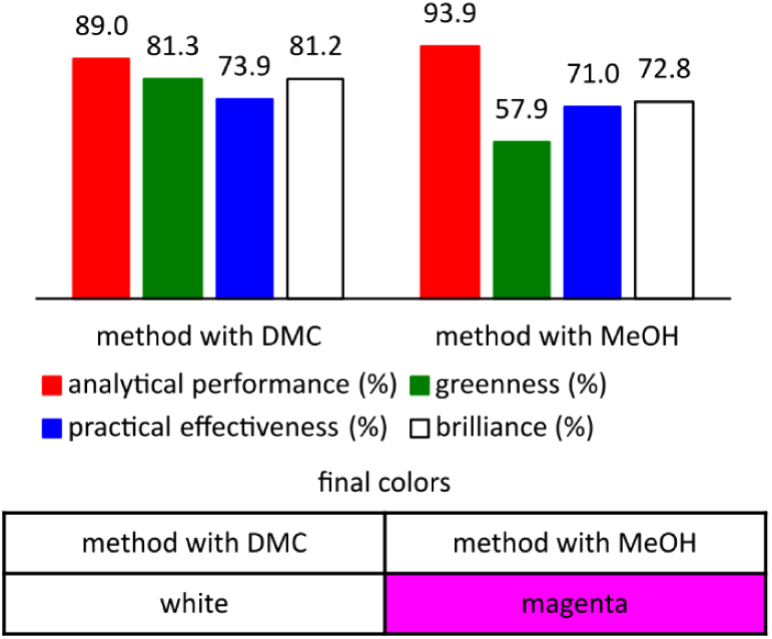
Results of whiteness evaluation of the developed methods obtained with the RGB tool.

The last tool was chosen to include in the comparison aspects the greenness, efficiency and economy of the developed methods. To perform this, the RGB tool was selected. Comparing analytical performance for the developed methods, both precision and accuracy yielded similar results, the range of linearity and sensitivity was assessed more favorably for the methanol method, which is mainly due to the use of larger injection volumes in this method than in the DMC method. The final result reached a value 5% points higher for the method employing methanol (94%) versus DMC (89%), with every parameter exceeding the “satisfactory value” (>66.6%). For the green assessment, the focus was on comparing the volumes of organic solvents that were required, their harmfulness, the amount of waste generated, and energy consumption. The method using DMC achieved above “satisfactory values” in each of these four criteria, as the consumption of this reagent was low, which also translated into a significant reduction in waste overall, and the harmfulness of this compound is only related to its flammability. In addition, the UHPLC technique does not require significant energy input. This resulted in a final rating of 81%. The reference method, due to the application of methanol, did not meet the conditions needed to exceed the “satisfactory value” for the three criteria, but their values are within the “tolerance range”. Energy consumption was evaluated as satisfactorily as for the greener method. This resulted in a final rating of 58%. The practical efficiency ratings of the two methods are similar, at 74% for the DMC method and 71% for the MeOH method (each of the compared parameters received values above satisfactory). Cost-effectiveness, sample throughput and sample consumption were compared in this criterion. Sample consumption is lower for the greener procedure because the UHPLC technique allows for a smaller injection volume and consumes considerably less mobile phase (due to a lower flow rate). However, dimethyl carbonate, compared to methanol, has more than double the price which is disadvantageous from an economic point of view, but not in a conclusive way, since it takes much smaller amounts to achieve the same effect. Sample throughput, on the other hand, received the same ratings. The above evaluations lead to a final result qualifying the method with DMC as white, which means it is a good candidate of choice for all applications. In contrast, the method with MeOH received a final score corresponding to magenta which means that it is a method of choice if no greener alternative is available. A chart comparing the different results and the final color rating of the methods is shown in Figure 5. The detailed evaluation of each parameter and the values assigned to each criterion developed for each of the four tools used are presented in the Supporting Information.

## Conclusions

An environmentally friendly UHPLC method using dimethyl carbonate as the sole organic solvent was developed for the first time both as an eluent in solid phase extraction and for the simultaneous determination of theobromine and caffeine in tea extracts. The developed methods demonstrated high sensitivity, linearity (R^2^ > 0.999), and precision (RSD < 2%). Recovery rates of 97–101% confirmed the accuracy of both methods. To accurately compare the effectiveness of DMC, a standard method was applied to extract theobromine and caffeine from teas and their subsequent determination based on the procedure for purifying the extracts obtained from the manufacturer and data from the articles. Their greenness and whiteness were evaluated. DMC, as a less harmful solvent compared to methanol, significantly improved the environmental profile of the method according to AGREEprep, AGREE, ChlorTox and RGB tool evaluations. Also, the change from HPLC to UHPLC contributed to better evaluations from these tools since it enabled a reduction in waste generation, saved solvents and energy, which highlighted the cumulative environmental benefits that can be achieved by modifying the method overall. However, it is important to note that DMC’s limited miscibility with water does not pose a difficulty for the elution of moderately polar compounds (such as methylxanthines) but prevents its application in the analysis of highly hydrophobic compounds and can pose a challenge for gradient elution, potentially affecting overall performance in more complex separations. Despite these limitations, the RGB tool showed equally high practical effectiveness and similar analytical performance of this more environmentally friendly method in relation to the developed reference method. The results highlight the potential of dimethyl carbonate to replace conventional organic solvents, such as methanol, without compromising the quality and effectiveness of analytical procedures.

## Supporting Information Available

Detailed evaluation of each parameter with its assigned value carried out using AGREEprep, AGREE and RGB tools for the developed methods. Calculations required to determine ChlorTox values (PDF)
