# Supplementary material for: Greener and Whiter Analytical Procedure for Theobromine and Caffeine Determination in Tea Using Dimethyl Carbonate as an Extraction Solvent and Mobile Phase Constituent in Reversed-Phase Liquid Chromatography
Source: ACS Omega. 2025 Mar 24;10(12):12432–40. doi: 10.1021/acsomega.4c11625 (PMC11966322; doi:10.1021/acsomega.4c11625)
Supplement: Supplementary file 1 — ao4c11625_si_001.pdf [file ao4c11625_si_001.pdf]

Supporting Information

Greener and Whiter Analytical Procedure for Theobromine and Caffeine Determination in Tea Using Dimethyl Carbonate as an Extraction Solvent and Mobile Phase Constituent in Reversed-Phase Liquid Chromatography

Oktawia Kalisz[1], Martina Catani[2], Szymon Bocian[1,*]

[1] Department of Environmental Chemistry and Bioanalytics, Faculty of Chemistry, Nicolaus Copernicus University, 7 Gagarin St., 87-100 Toruń, Poland;

[2] Department of Chemical, Pharmaceutical and Agricultural Sciences, University of Ferrara, via L. Borsari 46, Ferrara 44121, Italy

1. Greenness evaluation of sample preparation step for compared methods

The Table S1 and Table S2 show the criteria that the AGREEprep tool takes into account when assessing greenness, the data that was entered/selected for these criteria, and the greenness rating that the entered data generated, along with the final rating of the methods.

Table S1. A detailed evaluation of each parameter with its assigned value performed using the AGREEprep tool for the dimethyl carbonate-employed method.

Criterium of the AGREEprep calculations [1]	Input data	Obtained score	Color
1. Sample preparation placement	*ex situ*	0.0	red
2. Hazardous materials	0.0	1.0	green
3. Sustainability, renewability and reusability of materials	25–50% of reagents and materials are sustainable or renewable	0.25	orange
4. Waste	7.5	0.31	orange
5. Size economy of the sample	0.1	1.0	green
6. Sample throughput	24	0.75	green
7. Integration and automation	≤ 2 steps; semi-automated systems	0.75	yellow
8. Energy consumption	< 10 Wh per sample	1.0	green
9. Post-sample preparation configuration for analysis	liquid chromatography	0.25	orange
10. Operator's safety	1 hazard	0.75	green
Overall AGREEprep score	–	0.67	green

Table S2. A detailed evaluation of each parameter with its assigned value performed using the AGREEprep tool for the methanol-employed method.

Criterium of the AGREEprep calculations [1]	Input data	Obtained score	Color
1. Sample preparation placement	*ex situ*	0.0	red
2. Hazardous materials	5.5	0.0	red
3. Sustainability, renewability and reusability of materials	25–50% of reagents and materials are sustainable or renewable	0.25	orange
4. Waste	14.5	0.20	orange
5. Size economy of the sample	0.1	1.0	green
6. Sample throughput	12	0.62	green
7. Integration and automation	≤ 2 steps; semi-automated systems	0.75	yellow
8. Energy consumption	< 10 Wh per sample	1.0	green
9. Post-sample preparation configuration for analysis	liquid chromatography	0.25	orange
10. Operator's safety	3 hazards	0.25	orange
Overall AGREEprep score	–	0.42	orange

2. Greenness evaluation of analytical procedure for compared methods

The Table S3 and Table S4 show the principles that the AGREE tool takes into account when assessing greenness, the data that was entered/selected for these criteria, and the greenness rating that the entered data generated, along with the final rating of the methods.

Table S3. A detailed evaluation of each parameter with its assigned value performed using the AGREE tool for the dimethyl carbonate-employed method.

Principles for the AGREE calculations [2]	Input data	Obtained score	Color
1. Sample pretreatment activities	off-line analysis	0.48	yellow
2. Sample size	0.1	1.0	green
3. Location of the analytical device toward the investigated object	at-line	0.33	orange
4. Integration of analytical processes	3 or fewer	1.0	green
5. Automation and miniaturization of the sample preparation step	semi-automated; not miniaturized	0.25	orange
6. Presence of derivatization agents	0.0	1.0	green
7. Waste	7.5	0.42	yellow
8. Sample throughput	2 analytes; 12 analysis per hour	0.72	green
9. Energy consumption	UPLC	1.0	green
10. Origin of reagents	some reagents are bio-based	0.5	yellow
11. Toxicity of reagents and solvents	no	1.0	green
12. Operator's safety	highly flammable	0.8	green

Overall AGREE score	-	0.71	green

Table S4. A detailed evaluation of each parameter with its assigned value performed using the AGREE tool for the methanol-employed method.

Principles for the AGREE calculations [2]	Input data	Obtained score	Color
1. Sample pretreatment activities	off-line analysis	0.48	yellow
2. Sample size	0.1	1.0	green
3. Location of the analytical device toward the investigated object	at-line	0.33	orange
4. Integration of analytical processes	3 or fewer	1.0	green
5. Automation and miniaturization of the sample preparation step	semi-automated; not miniaturized	0.25	orange
6. Presence of derivatization agents	0.0	1.0	green
7. Waste	14.5	0.34	orange
8. Sample throughput	2 analytes; 12 analysis per hour	0.72	green
9. Energy consumption	liquid chromatography	0.5	yellow
10. Origin of reagents	some reagents are bio-based	0.5	yellow
11. Toxicity of reagents and solvents	yes; 5.5	0.32	orange
12. Operator's safety	highly flammable	0.8	green
Overall AGREE score	-	0.6	green

For both AGREEprep and AGREE tools, for some criteria, equations were presented in the articles to assess the greenness of a particular criterion. Thus, in order to provide a numerical value for evaluating a given criterion, the appropriate calculations were made.

3. Greenness evaluation of solvents (and their amounts) used in compared methods with the use of ChlorTox Scale

To enable the method to be evaluated using the ChlorTox scale, a simple model for quantifying overall chemical hazard, called the Weighted Hazard Number (WHN), was used, as presented in the original article [3]. In this approach, the overall hazard of the substance-of-interest (CH_{sub}) is determined as the sum of the hazards identified in the Section 2 of the safety data sheets (GHS format), with weights reflecting the degree of potential danger: 1 for category 1, 0.75 for category 2, 0.5 for category 3 and 0.25 for category 4. Therefore, the overall harmfulness of a given reactant is calculated from the formula:

$$CH_{sub} = 1 \cdot N_{cat1} + 0.75 \cdot N_{cat2} + 0.5 \cdot N_{cat3} + 0.25 \cdot N_{cat4}.$$

Since in the ChlorTox scale, chloroform plays the role of a universal standard for estimating the relative hazard of other chemicals, the calculated CH_{sub} value is divided by the CH value previously calculated for chloroform and equal to 5.75:

$$\text{WHN value} = \frac{CH_{sub}}{CH_{CHCl_3}} = \frac{CH_{sub}}{5.75}.$$

Then the volume of solvent used should be converted to mass (m_{sub}) and using the equation below, calculate the ChlorTox value:

$$\text{ChlorTox value} = \frac{WHN\ value \cdot m_{sub}}{1000}.$$

The harmfulness of the solvents used in developed methods and the individual results of the calculations aiming at the ChlorTox value are summarized in the Table S5.

Table S5. Comparison of the two developed methods in terms of relative risks with respect to chloroform (CH_{sub}/CH_{CHCl3}) obtained using the WHN model, in terms of the mass of individual reagents used per analysis (m_{sub}), and in terms of ChlorTox values indicating the degree of their chemical risk.

	Hazard categories of solvent*	CH_{sub}	WHN	m_{sub} [mg]	ChlorTox value [g]
Method with DMC	Category 2 (flammable liquid)	0.75	0.13	307.6	0.04
Method with MeOH	Category 2 (flammable liquid)<br>Category 3 (acute toxicity, oral)<br>Category 3 (acute toxicity, dermal)<br>Category 3 (acute toxicity, inhal.)<br>Category 1 (specific target organ toxicity - single exposure)	3.25	0.57	4350.5	2.46

* data obtained from the safety data sheet of the Carl Roth company

4. Whiteness evaluation of compared methods with the use of RGB tool

To compare the whiteness of the methods presented RGB tool was used [4]. The evaluation is carried out using Excel sheets (specifically designed by the authors) and customized by the users to the particular specifications of the methods. Since it is a highly flexible tool, it is first necessary to define the criteria relevant to the methods and to define reference values for each criterion: the "lowest acceptable value" (LAV), which corresponds to the score of 33.3, and the "lowest satisfactory value" (LSV), which corresponds to the score of 66.6. Four criteria were chosen to evaluate analytical performance colored in red: precision, accuracy, linearity range

and sensitivity. For ecological evaluation, colored in green, the focus was on comparing the amount of organic solvents required, their harmfulness, the amount of waste generated and energy consumption. The economic aspect colored in blue, included a comparison of cost-effectiveness, sample throughput and sample consumption. LAV and LSV values were selected based on data found during the literature review. The criteria used to evaluate analytical performance, greenness, and practicality were selected based on suggestions provided by the tool's authors in the article (after considering their alignment with the compared methods). The values selected as reference values for each criterion, the weighting of these criteria, and the ratings that were assigned to the developed methods along with their final scores are presented in Figure S1 and Figure S2.

method with dimethyl carbonate

		w=3			w=3			w=2		w=2	
REDNESS (analytical performance)	W=1	Accuracy (Recovery)			Precision (RSD%)			Linearity range		Sensitivity (LOD)	
CS: 89.0%	LAV=33.3	80-120%			10%			1 order (1-10)		5 µg/mL	
	LSV=66.6	90-110%			5%			2 orders (1-100)		1 µg/mL	
	Result	98-99%			2%			0.2-25		0.1 µg/mL	
	Score (0-100)	90	90	90	90	90	90	90	90	85	85
		w=3			w=3			w=2		w=2	
GREENNESS (safety and eco-friendliness)	W=1	Amount of chemicals			Harmfulness of chemicals			Amount of total waste		Energy consumption	
CS: 81.3%	LAV=33.3	10 mL/sample			4 hazard pictograms in total			50 mL/sample		moderate	
	LSV=66.6	1 mL/sample			2 hazard pictograms in total			10 mL/sample		low	
	Result	0.6 mL DMC			1 hazard pictogram			7.5 mL		very low	
	Score (0-100)	77	77	77	90	90	90	77	77	80	80
		w=4				w=4				w=2	
BLUENESS (productivity / practical effectiveness)	W=1	Cost-effectiveness				Sample throughput				Sample consumption	
CS: 73.9%	LAV=33.3	low				low				high	
	LSV=66.6	high				high				low	
	Result	moderately high				moderately high				very low	
	Score (0-100)	70	70	70	70	75	75	75	75	80	80
FINAL COLOR:		REDNESS		GREENNESS		BLUENESS		BRILLIANCE (MB):		81.2%	
WHITE		≥33.3%	≥66.6%	≥33.3%	≥66.6%	≥33.3%	≥66.6%				
		yes	yes	yes	yes	yes	yes				
Short annotation: 81.2white		Long annotation: 81.2white(89.0/1red-81.3/1green-73.9/1blue)									

Figure S1. Evaluation of the procedure using dimethyl carbonate as a solvent during SPE purification and chromatographic analysis.

method with methanol

		w=3			w=3			w=2		w=2	
REDNESS (analytical performance)	W=1	Accuracy (Recovery)			Precision (RSD%)			Linearity range		Sensitivity (LOD)	
CS: 93.9%	LAV=33.3	80-120%			10%			1 order (1-10)		5 µg/mL	
	LSV=66.6	90-110%			5%			2 orders (1-100)		1 µg/mL	
	Result	97-101%			2%			0.05-25		0.02 µg/mL	
	Score (0-100)	90	90	90	90	90	90	100	100	100	100
		w=3			w=3			w=2		w=2	
GREENNESS (safety and eco-friendliness)	W=1	Amount of chemicals			Harmfulness of chemicals			Amount of total waste		Energy consumption	
CS: 57.9%	LAV=33.3	10 mL/sample			4 hazard pictograms in total			50 mL/sample		moderate	
	LSV=66.6	1 mL/sample			2 hazard pictograms in total			10 mL/sample		low	
	Result	5.5 mL MeOH			3 hazard pictograms			14 mL		very low	
	Score (0-100)	50	50	50	50	50	50	65	65	80	80
		w=?	w=?	w=?	w=?	w=?	w=?	w=?	w=?	w=?	w=?
BLUENESS (productivity / practical effectiveness)	W=1	Cost-effectiveness				Sample throughput				Sample consumption	
CS: 71.0%	LAV=33.3	low				low				high	
	LSV=66.6	high				high				low	
	Result	moderately high				moderately high				moderate	
	Score (0-100)	80	80	80	80	75	75	75	75	50	50
FINAL COLOR:		REDNESS		GREENNESS		BLUENESS		BRILLIANCE (MB):		72.8%	
MAGENTA		≥33.3%	≥66.6%	≥33.3%	≥66.6%	≥33.3%	≥66.6%				
		yes	yes	yes	no	yes	yes				
Short annotation: 72.8magenta		Long annotation: 72.8magenta(93.9/1red-57.9/1green-71.0/1blue)									

Figure S2. Evaluation of the procedure using methanol as a solvent during SPE purification and chromatographic analysis.

References

[1] W. Wojnowski, M. Tobiszewski, F. Pena-Pereira, E. Psillakis, AGREEprep – Analytical greenness metric for sample preparation, TrAC - Trends in Analytical Chemistry 149 (2022) 116553. https://doi.org/10.1016/j.trac.2022.116553.

[2] F. Pena-Pereira, W. Wojnowski, M. Tobiszewski, AGREE - Analytical GREEnness Metric Approach and Software, Anal Chem 92 (2020) 10076–10082. https://doi.org/10.1021/acs.analchem.0c01887.

[3] P.M. Nowak, R. Wietecha-Posłuszny, J. Płotka-Wasylka, M. Tobiszewski, How to evaluate methods used in chemical laboratories in terms of the total chemical risk? – a ChlorTox Scale, Green Analytical Chemistry 5 (2023). https://doi.org/10.1016/j.greeac.2023.100056.

[4] P.M. Nowak, P. Kościelniak, What color is your method? adaptation of the rgb additive color model to analytical method evaluation, Anal Chem 91 (2019) 10343–10352. https://doi.org/10.1021/acs.analchem.9b01872.
